# Isochronal recovery behaviour on electromagnetic properties of polycrystalline nickel zinc ferrite (Ni_0.5_Zn_0.5_Fe_2_O_4_) prepared via mechanical alloying

**DOI:** 10.1038/s41598-021-99236-6

**Published:** 2021-10-04

**Authors:** Nor Hapishah Abdullah, Muhammad Syazwan Mustaffa, Mohd Nizar Hamidon, Farah Nabilah Shafie, Ismayadi Ismail, Idza Riati Ibrahim

**Affiliations:** 1 Functional Devices Laboratory (FDL), Institute of Advanced Technology, Universiti Putra Malaysia, 43400 Serdang, Selangor Malaysia; 2 Material Synthesis Characterization Laboratory (MSCL), Institute of Advanced Technology, Universiti Putra Malaysia, 43400 Serdang, Selangor Malaysia; 3 Centre of Pre-University Studies, Universiti Malaysia Sarawak, Kota Samarahan, Malaysia

**Keywords:** Materials science, Physics

## Abstract

A new approach through heat treatment has been attempted by establishing defects by the process of quenching towards electrical and magnetic properties in the nickel zinc ferrite (Ni_0.5_Zn_0.5_Fe_2_O_4_) sample. The measured property values in permeability and hysteresis characteristic gave their recovery behaviour in which the values, after quenching were recovered after undergoing the annealing. Interestingly, a different trend observed in the permittivity value whereas the value was increased after quenching and subsequently recovered after annealing. The mechanisms which produced the changes is believed to be involved by defects in the form of vacancies, interstitials, microcracks and dislocations created during quenching which gave rise to changes in the values of the complex permeability and permittivity components and hysteresis behaviour.

## Introduction

A remarkable magnetic nanomaterial has been well known to be very stable in term of fundamental and various applications such as microwave absorbers, memory devices, microwave devices, environmental sensor, gas sensor, supercapacitor, magnetic hyperthermia cancer treatment, agents in drug delivery, magnetic resonance imaging, ferrofluids and also magnetic refrigeration. Particle size, morphology, cation distribution, compositions, core shell structure and structural characteristic effect has known to greatly influence the magnetic properties of the nanomaterial. Recently, the magnetic properties have been improved via synthetic techniques which have opened a wide range of applications^1–19^. As for in electronic application, devices based on polycrystalline Ni_0.5_Zn_0.5_Fe_2_O_4_ have been comprehensively applied due to their remarkable high resistivity, chemical stability, mechanical hardness and its high permeability value^20^. Microstructure elements such as grain size, grain boundary and pores were known to be highly impacted on the magnetic and electrical properties in Ni_0.5_Zn_0.5_Fe_2_O_4_ through the chosen synthesis process^21^. Conventional ceramic techniques based on oxide materials followed by sintering temperature at 1000 °C were normally used to synthesize ferrite with superior properties^22^. The extensive use of Ni_0.5_Zn_0.5_Fe_2_O_4_ and the general knowledge of their physical properties mainly contributed by their preparation condition have inspired us to investigate the heat treatment effect on complex permeability and permittivity along with hysteresis behavior in nickel zinc ferrite. The stoichiometric composition of Ni_0.5_Zn_0.5_Fe_2_O_4_ also has been known to have an effect on the electrical and magnetic properties of ferrites^23^. The nickel–zinc ferrite with the composition of Ni_0.5_Zn_0.5_Fe_2_O_4_ has been chosen in this study due to its chemical stability, high resistivity, noble soft magnetic property and low dielectric loss. As for recovery process, it is known as the behavior of a material’s property whereas the initial values have been changed by cold-working or quenching tend to be recovered or restored after undergone a heat treatment process such as annealing through using the same annealing time during the recovery process^24–26^. In metals, the recovery behavior is usually attributed^27–29^ to a huge increment in atomic vacancies by performing quenching, while the gradual decrease in the amount of the vacancies occurred with respect to the reheating process by annealing with slow cooling rate. The recovery behavior in Ni_0.5_Zn_0.5_Fe_2_O_4_ as a test material in this research work is based on the same idea referring to the recovery process which normally conducted in metal materials. Currently, a study regarding to the introduction and removal of defects created at atomic level has not yet been explored by researchers in magnetic materials in order to alter or to enhance the magnetic properties. This has inspired us to study the possible effect of defects with respect to electromagnetic properties. Therefore, the effect of quenching and subsequent annealing in Ni_0.5_Zn_0.5_Fe_2_O_4_ sample towards its complex permeability and permittivity as well as its *B-H* hysteresis behavior has been investigated experimentally. Interestingly, the effect of the process was carried out by using only one sample subjected to multiple heat treatment process such as sintering, quenching and annealing.

## Materials and method

Raw materials used to prepare the stoichiometric mixture were nickel oxide (NiO), 99% (Alpha Aesar), zinc oxide (ZnO), 99% (Alpha Aesar) and iron oxide (Fe_2_O_3_), 99.8% (Strem Chemicals) weighed according to the calculated composition formula as shown in Eq. (1):

1 $$ 0.5{\text{NiO}} + 0.5{\text{ZnO}} + {\text{Fe}}_{2} {\text{O}}_{3} \to {\text{Ni}}_{0.5} {\text{Zn}}_{0.5} {\text{Fe}}_{2} {\text{O}}_{4} $$

**Figure 1:**
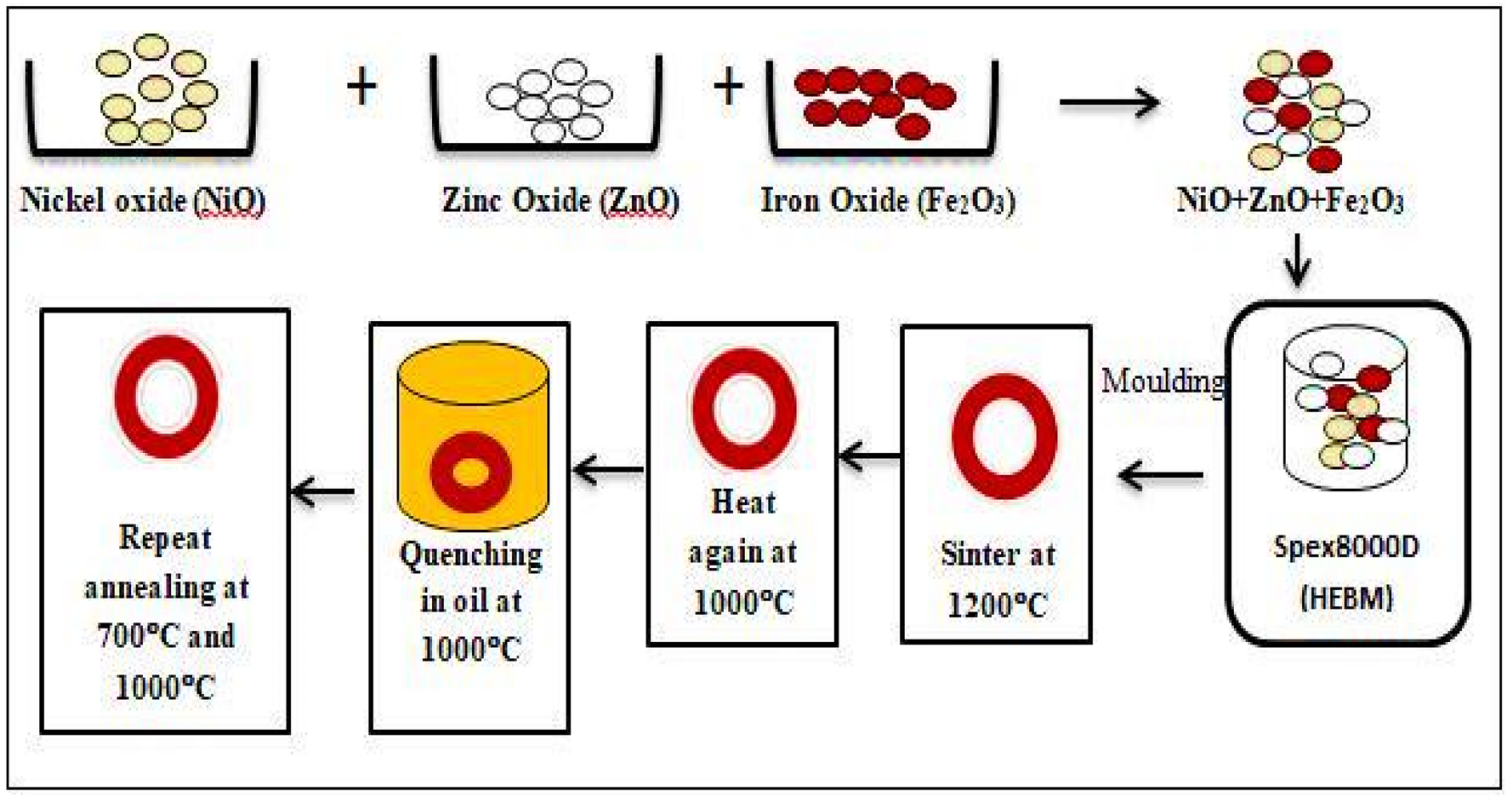
Schematic diagram of the synthesis of nickel zinc ferrite (Ni_0.5_Zn_0.5_Fe_2_O_4_) and subsequent heat treatment process.

**Figure 2:**
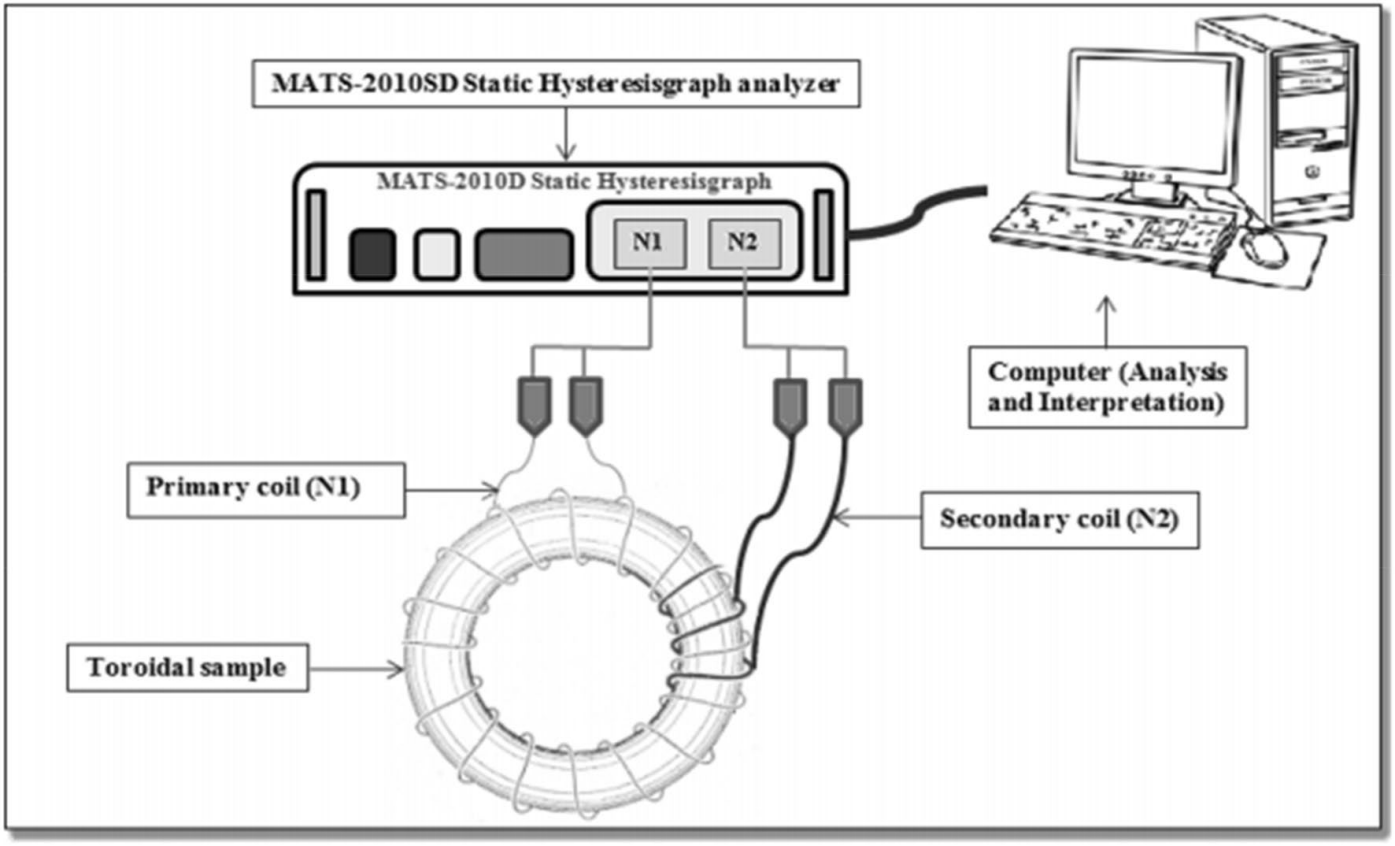
Schematic diagram of *B–H* hysteresis loop measurement^30^.

These powders were crushed by using a Spex8000D milling machine with a ball to powder ratio (BPR) of 10:1 for 3 h of milling time. Then, the powder was blended with approximately 1 wt.% of polyvinyl alcohol (PVA) as a binder and 0.3 wt.% of zinc stearate powder as a lubricant. The powder was moulded into toroidal shape using a hydraulic pressing machine under a pressure of 300 MPa. The single-toroidal sample was sintered at 1200 °C for 10 h in an ambient air atmosphere. Sample in toroidal form was coiled by using both copper wire and wrapped with 30 turns for primary coil (N1) and 60 turns for secondary coil (N2). Then, the *B–H* hysteresis loop characteristics of toroidal sample wired-wound were investigated via MATS-2010SD Static Hysteresisgraph analyzer under applied magnetic fields of 0 to 800 Oe (Fig. 1). The analyzer is connected to a computer and software used to attain and record the data (Fig. 2). The real complex permeability and complex permittivity were measured by using an AgilentHP4291B Impedance/Material Analyzer in the range of 1 MHz to 1.8 GHz attached with low and high impedance sample holder respectively**.** The structural analysis has been carried out by X-ray Diffraction (XRD) and Fourier Transform Infrared (FTIR) Spectroscopy. X-ray diffraction analysis has been carried out using CuKa radiation (Phillips Expert Pro PW3040) with 2θ (20–70). Perkin Elmer IR was used to record the Fourier Transform Infrared spectra of Ni_0.5_Zn_0.5_Fe_2_O_4_. The measurement of the recovery process in Ni_0.5_Zn_0.5_Fe_2_O_4_ sample on selected parameters such as (*μ*′, *μ*″, *ε*′, *ε*″, *B*_*s*_ and *H*_*c*_) has been carried out after sintering at 1200 °C. The quenching process was performed at room temperature by immersing the sample into a beaker containing cooking oil after 1000 °C heating with 2 h holding time and the selected parameters were measured. After that, the sample was annealed in air at 700 °C and 1000 °C for 2 h holding time and cooled slowly to room temperature. The selected parameters were then again measured after each annealing process at 700 °C and 1000 °C respectively. The detailed chemical reaction of the synthesized nanoparticles along with the schematic representation for the step-by-step synthesis of nanoparticles has been described in Fig. 1.

## Results and discussion

**Figure 3:**
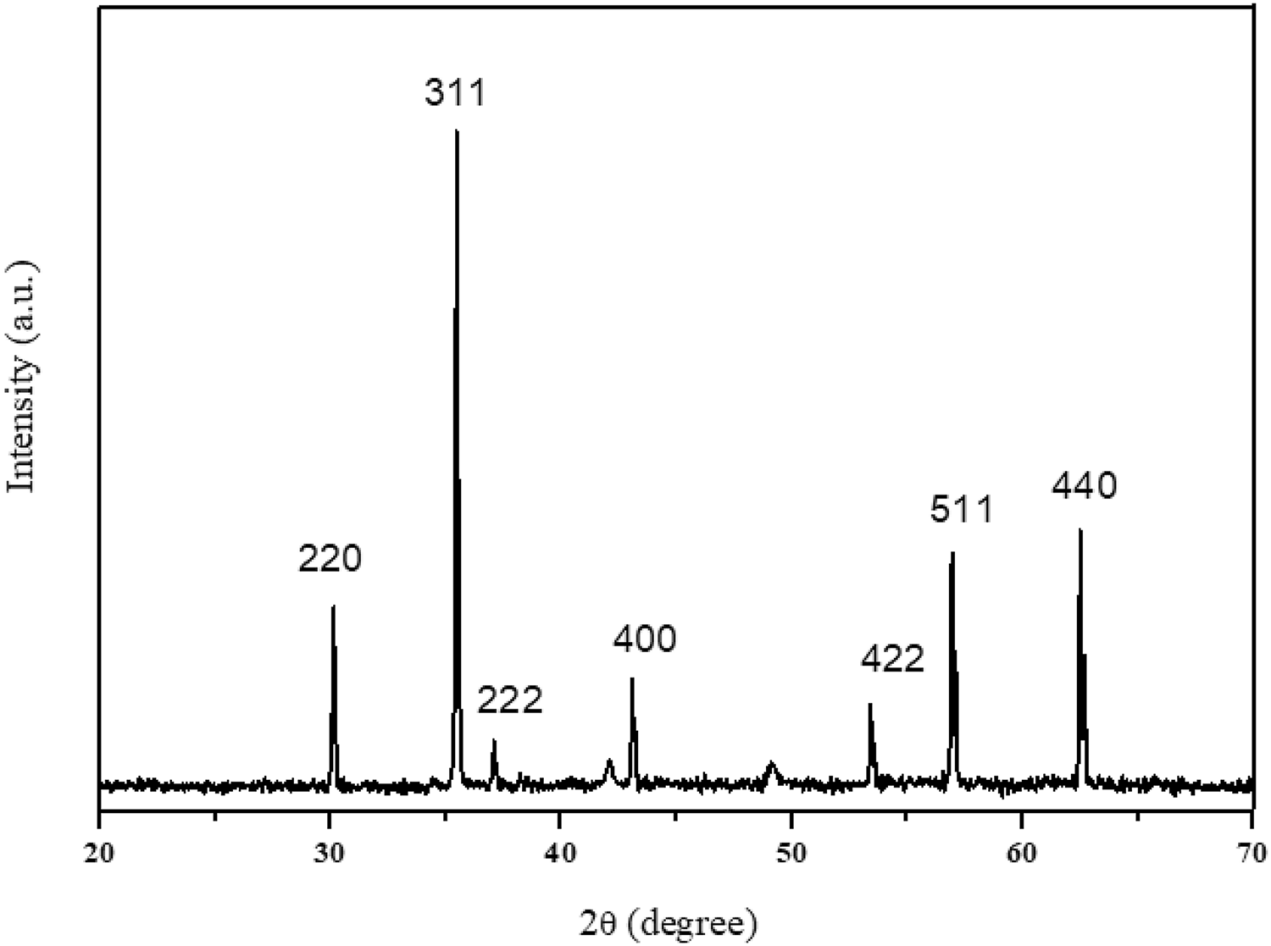
XRD pattern of nickel zinc ferrite (Ni_0.5_Zn_0.5_Fe_2_O_4_) sintered at 1200 °C.

Figure 3 shows the XRD analysis of Ni_0.5_Zn_0.5_Fe_2_O_4_ after undergone sintering at 1200 °C. As can be seen in the figure, all the peaks were corresponded to the single phase crystallization of Ni_0.5_Zn_0.5_Fe_2_O_4_ with no other extra planes corresponding to any extra phase of impurity were obtained indicating the formation of nickel–zinc ferrite. The result also was in a good agreement with previous researchers^31^. All peaks match the standard pattern of nickel–zinc ferrite (00–008-0234) of the ICDD database showing Ni_0.5_Zn_0.5_Fe_2_O_4_ and can be clearly indexed to the seven major peaks of the spinel ferrites, which are (220), (311), (222), (400), (422), (511) and (440) planes of a cubic unit cell, corresponding to spinel structure. The sharp and narrow XRD peak indicates a larger particle size and the enhancement of crystallinity.

**Figure 4:**
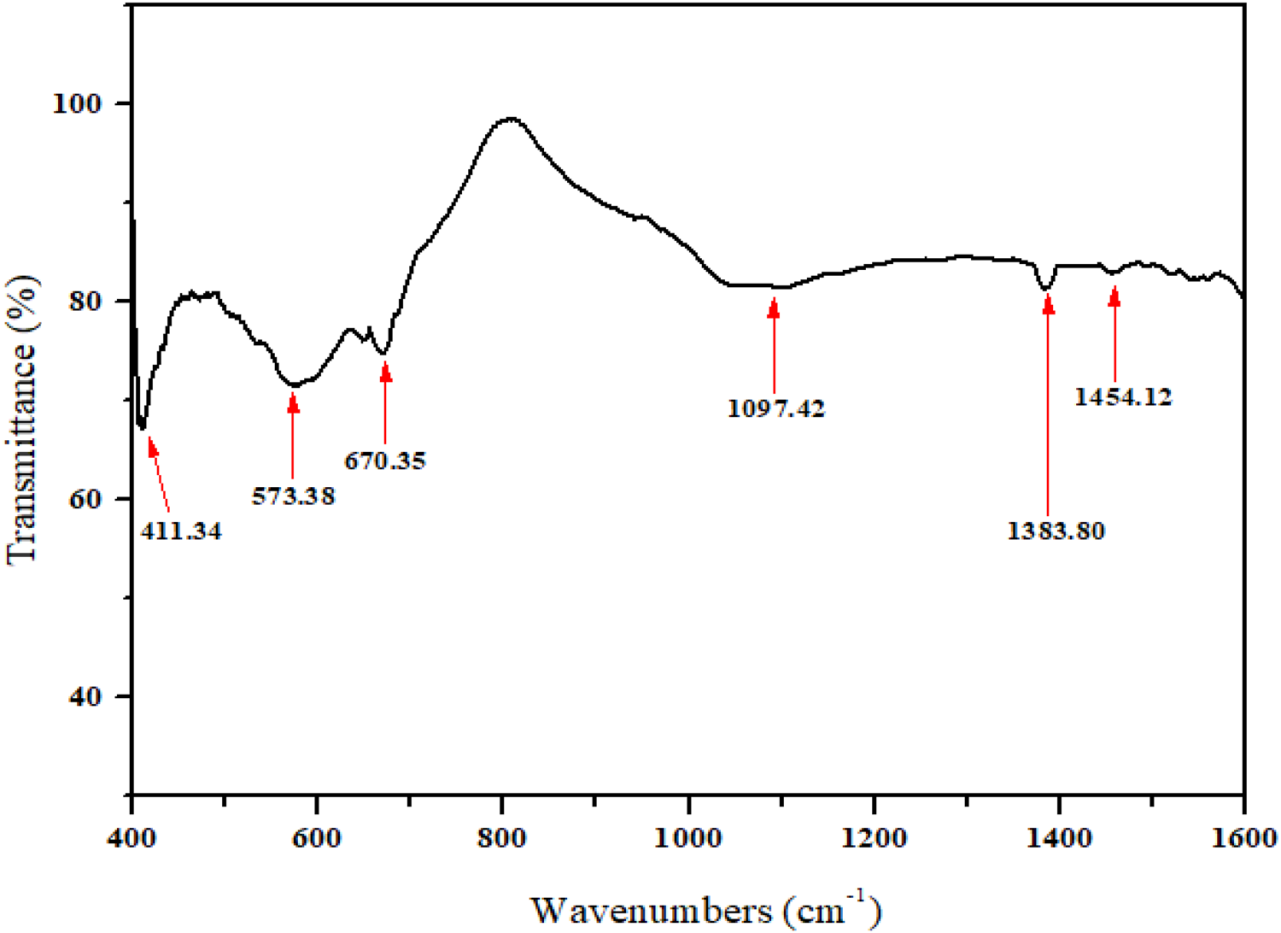
FTIR spectra of nickel zinc ferrite (Ni_0.5_Zn_0.5_Fe_2_O_4_) sintered at 1200 °C.

Figure 4 illustrates the FTIR spectra which help in confirming the formation of the spinel structure in Ni_0.5_Zn_0.5_Fe_2_O_4_ sample. The tetrahedral and octahedral positions are preferred by Zn and Ni atoms, respectively^32,33^. Fe^3+^ cations, on the other hand, can occupy both octahedral and tetrahedral sites. The spinel structure has two characteristic bands that are in the 400–600 cm^−1^ range^32,33^. At 573.38 cm^−1^ which is corresponding to intrinsic metal stretching vibrations at the tetrahedral site (Fe–O), meanwhile at 411.34 cm^−1^ is matches to octahedral–metal stretching (Ni–O and Zn–O). Other vibrations are represented by bands of much lower intensity. Peaks with wave numbers 1097.42 cm^−1^ and 1383.80 cm^−1^ are caused by the stretching vibration of the C–O bond. The C=O stretching vibration of the carboxylate group CO^2−^ ^34^ is correlated for the peak with wavenumber 1454.12 cm^−1^.

**Figure 5:**
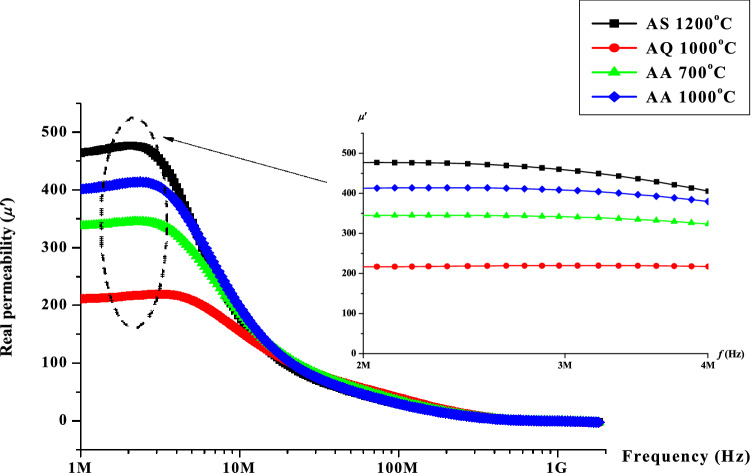
Graph of real permeability, *µ*′ versus frequency after sintering at 1200 °C, after quenching at 1000 °C, after annealing at 700 and 1000 °C.

Figure 5 show the real permeability, *µ*′ value as a function of frequency ranging from 1 MHz to 1 GHz for Ni_0.5_Zn_0.5_Fe_2_O_4_ sintered at 1200 °C followed by quenching at 1000 °C and annealing at 700 °C and 1000 °C respectively. The real permeability value was obviously decreased after quenching process has been performed at 1000 °C. The following annealing process at 700 °C and 1000 °C showed an increase in the real permeability values which were higher than after quenching. The permeability value observed at 1200 °C (AS1200°C) sintering temperature could be related to larger grain size with high density value^35–37^. Larger grain size with fewer amounts of grain boundaries at this sintering temperature would contribute to the mobility of domain walls hence increasing the permeability value. At larger grain, the reduced number of pores also contributed to the ease of domain wall motion due to less pining which act as a barrier in the sample. Higher sintering temperature will also affected on the decrement in magnetic anisotropy by reducing the internal stress and crystal anisotropy, hence reducing the interruption of the domain walls^40^. The similar trend also has been observed within the same material carried out by previous researchers^35–37^. Snoek’s Law has also proven that higher permeability would have a lower value of frequency as shown in Eq. (2) ^38^:

2 $$ f_{r} (\mu_{i} - 1) = \frac{4}{3\gamma Ms} $$

where *f*_*r*_ is resonance frequency, *μ*_*i*_ is initial permeability, γ is gyromagnetic ratio and *M*_*s*_ is saturation magnetization.

After quenching has been performed at 1000 °C, at this temperature, the atoms at their own position were vibrating and elevating out of their position due to thermal energy supplied through the temperature. A sudden thermal shock via quenching process has left the atoms to be trapped on other atoms and creates vacancies. The atoms could be stuck at the surface of the sample or at an interstitial place via being interrupted from its lattice position at equilibrium state. The atomic defects seem to be existed as the atoms could not return to its original position. Therefore, the magnetization will be reduced therefore affecting the permeability value to drop compared to the value before quenching has been carried out. Additionally, the presence of defects will increased distortion or strain of their surfaces due to domain walls pinning created after quenching which result in lower value of permeability. Conversely, after annealing process, the oxygen absorption rate was increased hence filling back the oxygen which increases the permeability value to be recovered. After annealing process at 700 °C, the value of permeability was observed to be increased due to a decrease of the magnetic anisotropy by decreasing the internal stress and crystal anisotropy, which reduces the domain walls interference hence increasing the permeability value of Ni_0.5_Zn_0.5_Fe_2_O_4_ sample. Subsequent annealing process performed at 1000 °C showed even better recovery compared to annealing at 700 °C. This is due to higher thermal energy supplied and higher rate of oxygen absorption which allow better recovery in Ni_0.5_Zn_0.5_Fe_2_O_4_ sample due to unpinning process in the domain wall motion. However, the value does not reach the actual value as the same in the early sintering at 1200 °C. This might be due to the amount of atom which requires longer holding time during annealing in order to allow the atom to return to their original position hence giving a higher value in the permeability.

**Figure 6:**
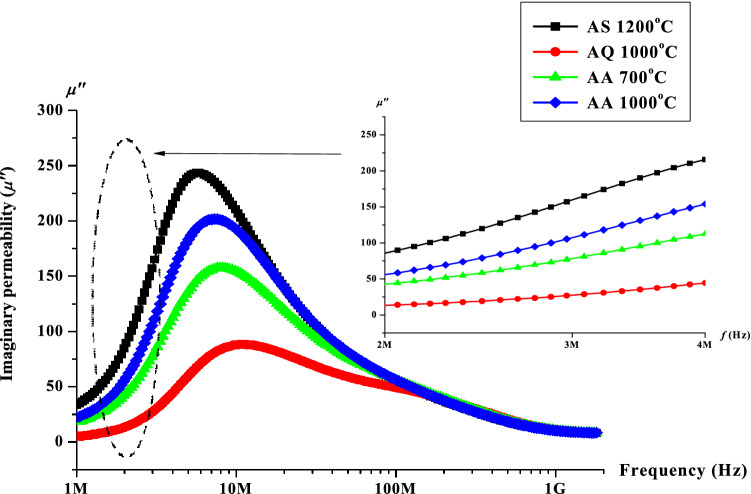
Graph of imaginary permeability, *µ*″ versus frequency after sintering at 1200 °C, after quenching at 1000 °C, after annealing at 700 and 1000 °C.

The graph of loss factor, *μ*″ as a function of frequency for the sample sintered at 1200 °C and after undergone quenching and annealing heat treatments are shown in Fig. 6. The same trend with real permeability is seen in the case of variation in loss factor with respect to frequency. The loss factor value was observed to decrease after quenching process at 1000 °C has been performed. The loss factor values after annealing at 700 °C and 1000 °C respectively were both recovered almost to its original value. The loss factor value was also known to depend on the effect of grain size. There is more domain walls existed in larger grain size. This will allow the domain walls to move at ease hence inducing an eddy current. Larger grain size will also tend to increase the amount of magnetic domain which contributes to loss due to delay in domain wall motion. After defect has been created via quenching process, the defects existed will obstruct the eddy current flow. The pinning effect in domain wall movement via defects created will impede eddy current flow resulting in slower domain wall movement hence giving a lower value of loss factor. Generally, the losses are associated with both spin rotation relaxation and domain-wall relaxation. A lower value of the loss factor was observed due to defects created after the quenching process. Subsequently, the samples annealed at 700 and 1000 °C revealed a recovery process whereas the loss factor value was recovered to its original value before quenching. It can be seen that the sample exhibited a similar characteristics as in the real permeability value in which the loss factor value was decreased after quenching and increased back after annealing at 700 °C and 1000 °C. Point defects such as vacancy and interstitial defects are believed to be formed after quenching has been performed. Moreover, the increased in strain and defect surfaces will contributed to a stronger domain wall pinning. Additional energy will be required in the form of applied field will be required in order to free them from pinning centres, hence giving a lower value of both real and imaginary permeability after quenching. After the annealing process at 700 °C and1000°C respectively, the atomic vacancies caused by quenching process will be refilled with returning displaced atoms as well as the reduction of defect surface strain hence resulting to higher value of the loss factor via the recovery of better domain wall movements. Comparing Figs. 5 and 6, it can be observed that, the frequency of the resonance region occurred first at *μ*′ and was later followed by *μ*″. The lag occurred was contributed by the real permeability is in phase with the external field while the imaginary permeability is out of phase with the external phase^39^. The behaviour of *μ*′ and *μ*″ can be qualitatively explained again by using Snoek’s Law as in Eq. (2)^40^. The details value of real (*µ*′) and imaginary (*µ*″) permeability is shown in Table 1.

**Table 1:** The real (*µ*′) and imaginary (*µ*″) permeability after sintering, quenching and after annealing at 3 MHz.

Process	Parameter	
	Real permeability (*µ*′)	Imaginary permeability (*µ*″)
After sintering at 1200 °C	475	150
After quenching at 1000 °C	215	18
After annealing at 700 °C	350	50
After annealing at 1000 °C	415	75

**Figure 7:**
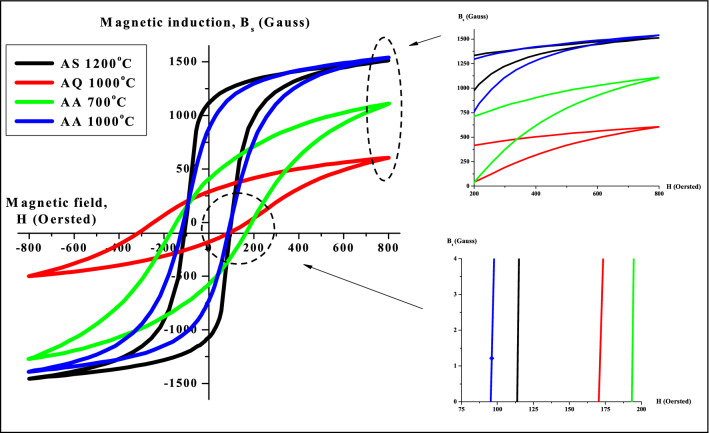
*B–H* hysteresis curve for sample after sintering at 1200 °C, after quenching at 1000 °C, after annealing at 700 and 1000 °C.

**Figure 8:**
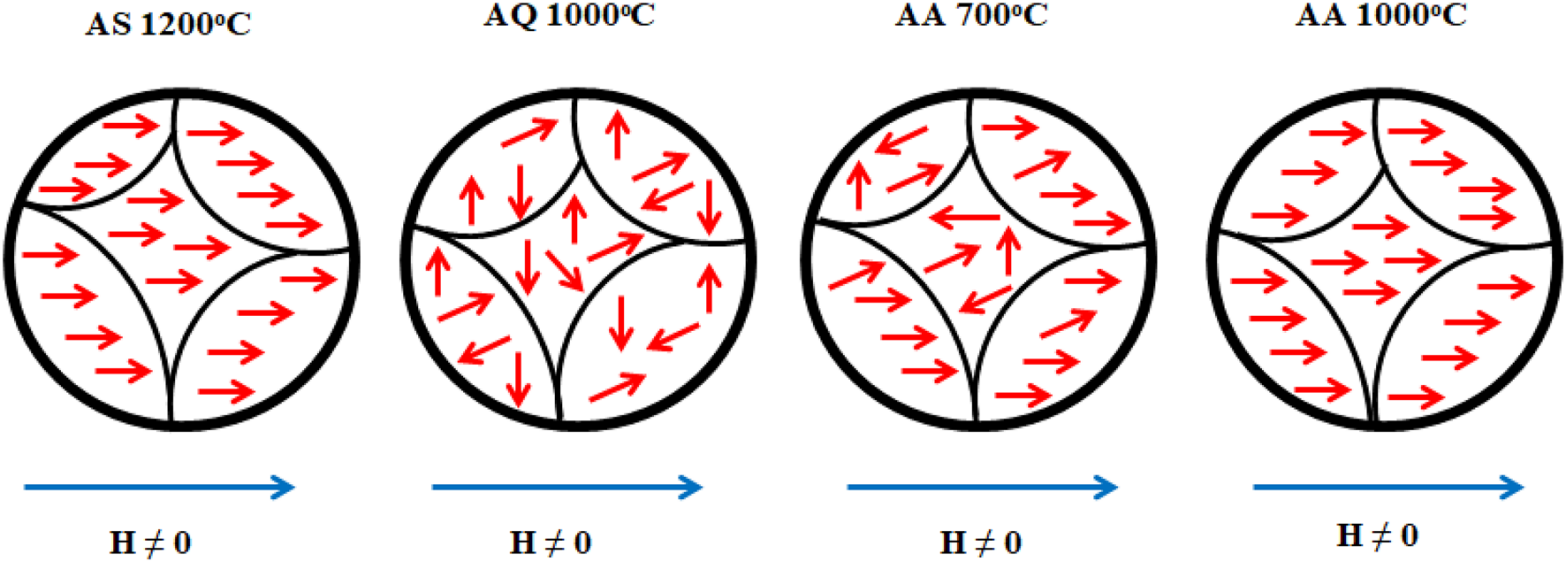
Domain wall prediction for sample after sintering at 1200 °C, after quenching at 1000 °C, after annealing at 700 and 1000 °C.

**Figure 9:**
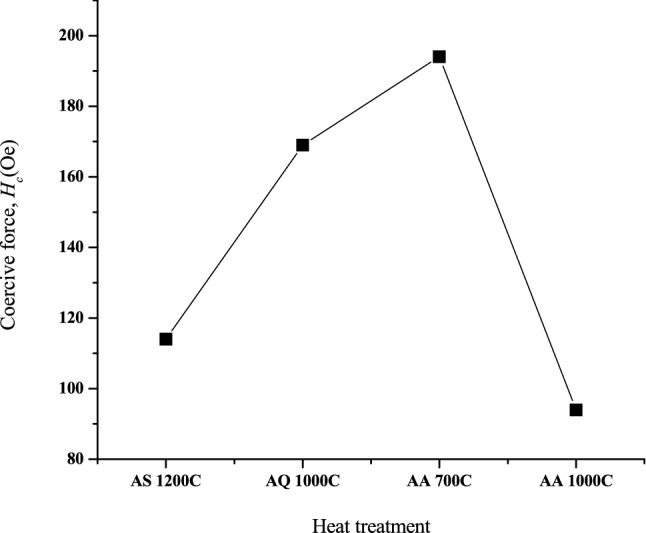
Coercive force, *H*_*c*_ versus heat treatment after sintering at 1200 °C, after quenching at 1000 °C, after annealing at 700 and 1000 °C.

Figure 7 depicts the *B–H* hysteresis loops of Ni_0.5_Zn_0.5_Fe_2_O_4_ sample after undergone sintering, quenching and annealing process. The measurements were taken at room temperature (~ 30 °C). The main parameters involved in *B-H* hysteresis are saturation induction (*B*_*s*_) and coercive field (*H*_*c*_). The hysteresis properties of Ni_0.5_Zn_0.5_Fe_2_O_4_ sample are highly affected by the structure and the fraction of single phase as well as the grain size^41^. As can be seen in Fig. 7, the hysteresis loops characteristics generally showed a change in magnetic phase from strong to moderate ferromagnetism and recovering back to strong ferromagnetism parallel with various heat treatments that is after sintering, after quenching and after annealing respectively. Initially after sintering at 1200 °C, the hysteresis loop behaves narrower, erected and well-defined sigmoid form. Due to their high volume fraction of the complete Ni_0.5_Zn_0.5_Fe_2_O_4_ phase and larger grain size, a strong ferromagnetism state is predominated at this stage. A higher *B*_*s*_ value observed after sintering at 1200 °C is a result of the gradual increased in crystallinity and grain size, as can be established in other ferrites^31,35–38,42,43^. Besides that, high sintering temperatures reduce magnetic anisotropy by decreasing crystal anisotropy and internal stress; thereby lower the hindrance to domain wall motion^35–38^. More domain walls exist in the grain as grain sizes increase. As a result, the domain walls can easily move in the larger grain. After quenching has been performed at 1000 °C, a slanted sigmoid shape consisting moderate ferromagnetic phase as well as paramagnetic phase can be observed. The paramagnetic phase was speculated to be appeared due to the domain wall pinning resulting from atomic level defects created during quenching. This will impact on the value of *B*_*s*_ which has been observed to be decreased after quenching has been performed. Domain wall pinning due to defects created at atomic level will complicate the spin inside the domain hence slowing down the ability of the domain wall to be saturated. The disturbance occurred in the *B*_*s*_ will results in the changes in the *B*_*s*_ value. Meanwhile, the *H*_*c*_ value of Ni_0.5_Zn_0.5_Fe_2_O_4_ sample after quenching has been performed was observed to be increased compared to after sintering. The increase in the *H*_*c*_ value was attributed to the atomic defects created during quenching. Higher energy is required in order to overcome the difficulty of domain wall movement which has been pinned by defects will contribute to higher *H*_*c*_^19,20^. After the first stage of annealing at 700 °C, the *B*_*s*_ value was recovered twice as much as after quenching has been performed. The recovery was contributed by the reduction of domain wall pinning effect allowing domain wall movement occur at better stage. Insufficient thermal energy supplied during annealing at 700 °C was not enough to reduce entirely the domain wall pinning due to atomic defects after quenching. Meanwhile, the *H*_*c*_ value after annealing at 700 °C was observed to be higher than after quenching. An atomic defect has resulted in incomplete rearrangements of the super-exchange interaction inside the Ni_0.5_Zn_0.5_Fe_2_O_4_ sample. The value was found to be higher due to the combination of residual atomic defect (disordered magnetic moment) as well as magnetic moment alignment which has been recovered due to the annealing process at 700 °C. As for after stage 2 annealing at 1000 °C, the *B*_*s*_ value was observed to fully recovered to its original value before quenching has been performed. This is due to sufficient thermal energy supplied at 1000 °C to alter or to remove the atomic defects due to quenching. The recovery also was contributed to better super-exchange interaction inside Ni_0.5_Zn_0.5_Fe_2_O_4_ sample hence giving better domain wall movement. Domain wall movement can also move easily at this stage whereas with minimum supplied magnetic field can easily magnetized the sample hence giving a lower value of *H*_*c*_. The *H*_*c*_ value was also recovered to almost close to its original value before quenching has been conducted. The lower value reveals the ease of magnetic moment to be aligned due to the removal of atomic defects. Detailed value of hysteresis parameters such as *H*_*c*_ and *B*_*s*_ of Ni_0.5_Zn_0.5_Fe_2_O_4_ sample are listed in Table 2. The domain wall movement after undergone heat treatments such as sintering, quenching and annealing are predicted as in Fig. 8. The effect of heat treatments towards *H*_*c*_ were also has been detailed in Fig. 9.

**Table 2:** The magnetic induction, *B*_*s*_ and coercive force, *H*_*c*_ after sintering, quenching and after annealing.

Process	Parameter	
	Magnetic induction (*B*_*s*_)	Coercive force (*H*_*c*_)
After sintering at 1200 °C	1500	114
After quenching at 1000 °C	500	169
After annealing at 700 °C	1000	194
After annealing at 1000 °C	1550	94

**Figure 10:**
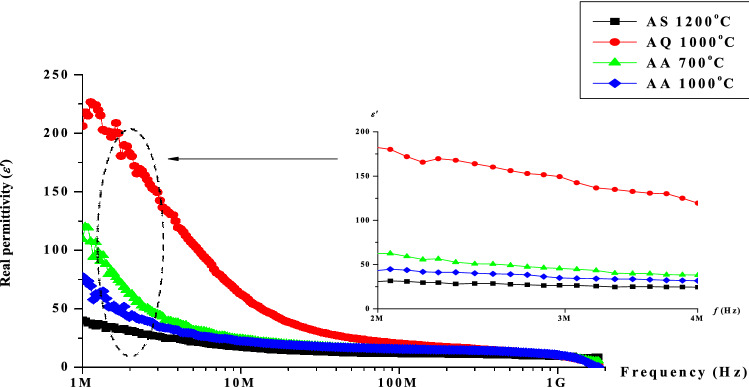
Graph of real permittivity, *ε*′ as a function of frequency after sintering at 1200 °C, after quenching at 1000 °C, after annealing at 700 and 1000 °C.

Dielectric permittivity or relative complex permittivity could be expressed as *ε*_*r*_ = *ε*′−*jε*″, where *ε*′ is real permittivity (capacity of the material to be polarized or the energy stored) while *ε*″ is imaginary permittivity (dissipated energy commonly in the form of heat. The *ε*′ value of Ni–Zn ferrite sample were observed in the frequency range from 1 MHz to 1 GHz at room temperature environment as revealed in Fig. 10 after undergone sintering (AS), quenching (AQ) and annealing (AA) respectively. Generally, the highest value of *ε*′ is observed at 1 MHz for all samples and the *ε*′ decreases with the increase of frequency due to the electron exchange between ferrous (Fe^2+^) and ferric (Fe^3+^) ions which does not follow the applied field, causing a reduction in the involvement of interfacial polarization. Thus, the *ε*′ decreases at higher frequency for all samples obeying a normal dielectric behaviour that have been studied by previous researchers^23,42–46^. This behaviour of ferromagnetic material might be due to the interfacial polarization as predicted by Maxwell and Wagner^47,48^. As for the effect of heat treatment towards *ε*′ as a function of frequency, the *ε*′ value exposed a sudden increased after quenching at 1000 °C has been performed. The atoms at their own position were vibrated and elevated out of their position due to thermal energy supplied through the temperature during quenching. A rapid cooling in the cooking oil via quenching left the atoms to be trapped on other atoms hence creating vacancies. The atoms could be dislocated at the surface of the sample or at an interstitial place via being interrupted from its lattice position at equilibrium state. The atomic defects are existed as the atoms could not return to its original position. Therefore, the *ε*′ value was observed to increase. Furthermore, the presence of defects will also increase distortion or strain of their surfaces promoting the mobility of Fe^2+^ ions hence giving higher value of the *ε*′. After annealing process at both 700 and 1000 °C, the *ε*′ values were recovered almost close to the same to the initial value (AS). The values obtained were caused by atomic defects removal during annealing process at both temperatures. However, the value do not fall exactly the same to the initial value of *ε*′ due to the remnants of the remaining defects. Annealing process was known to recover the bonding between Fe^2+^ and O^2−^ hence reducing the amount of Fe^2+^ and Fe^3+^ ions which contribute to lower values of *ε*′.

**Figure 11:**
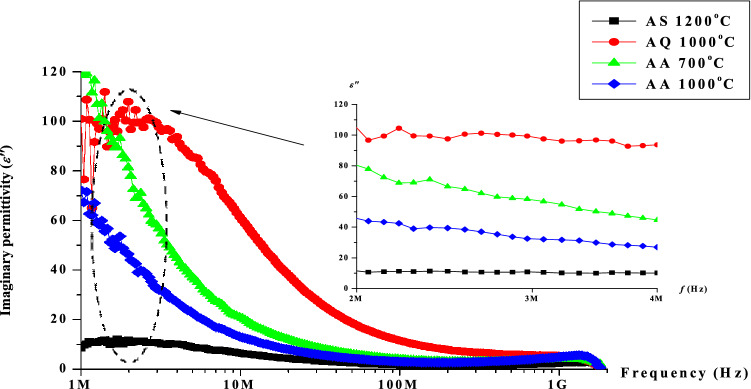
Graph of imaginary permittivity, *ε*″ as a function of frequency after sintering at 1200 °C, after quenching at 1000 °C, after annealing at 700 and 1000 °C.

Figure 11 shows the variation in imaginary permittivity (***ε***″) of Ni–Zn ferrite sample after undergone sintering at 1200 °C followed by quenching at 1000 °C and annealing at 700 °C and 1000 °C respectively. Generally, the trend of the values of *ε*″ decrease with the increase of frequency after undergone heat treatments. Theoretically, the *ε*″ value arises due to the lag in molecular polarization due to a moving electric field in a dielectric material. The dipole has sufficient time to reach equilibrium at lower frequency before the field changed. Specifically, when the relaxation process is rapid compared to the frequency of applied field, losses are small. Meanwhile at higher frequency, when the dipole orientation could not follow the applied field, the absorption of energy will be dissipated. The loss behaviour is analogous to that of the real permittivity whereas the values of the imaginary permittivity decrease with the increase of the frequency. The decrease take place when the jumping frequency of electric charge carriers could not follow the alternation of applied field^49^. The value *ε*″could be governed by on a number of factors such as Fe^2+^ content and homogeneity, which in turn depends on the composition and sintering temperature of the samples. A similar trend is observed after heat treatment of quenching and annealing process in the case of imaginary permittivity. The value was observed to be increased evidently after quenching has been performed and return to its almost original value after annealing has been carried out. The higher value after quenching was contributed by the more number of Fe^2+^ ions on B sites as mentioned in previous research work^50^. This is due to the atomic defect created during quenching process. Quenching has affected on the Fe^2+^ and Fe^3+^ mobility hence giving higher value of *ε*″. After annealing process, the atomic defects have been removed and recovered hence having less Fe^2+^ and Fe^3+^ in the sample. However, the value again does not fall exactly the same as the initial due to the remnant atomic defects created during quenching. Details of the real and imaginary permittivity value after undergone sintering, quenching and annealing at 3 MHz has been indicated in Table 3.

**Table 3:** The real (*ε*′) and imaginary (*ε*″) permittivity after sintering, quenching and after annealing at 3 MHz.

Process	Parameter	
	Real permittivity (*ε*′)	Imaginary permittivity (*ε*″)
After sintering at 1200 °C	28	10
After quenching at 1000 °C	60	100
After annealing at 700 °C	48	60
After annealing at 1000 °C	40	40

## Conclusions

The empirical recovery behavior in permeability, hysteresis characteristics and permittivity with respect to the heat treatment sequence in Ni_0.5_Zn_0.5_Fe_2_O_4_ sample was discovered after each sequence of sample sintering, quenching and annealing respectively. The complex permeability and hysteresis curve of the nickel zinc ferrite sample showed a recovery behavior whereas the values were decreased due to the domain wall pinning after defect formation via quenching and increased back after annealing. The values were reduced. Contrarily in the permittivity value, the recovery behavior was observed for permittivity values in which the value of the real permittivity was increased even higher than before quenching via defect formation due to the mobility of the Fe^2+^ hence giving higher value of dielectric permittivity. Since the lowering and recovering of the permeability, hysteresis parameters and permittivity components occurred without any grain growth, it can be resolved that the changes were related with the introduction and removal of atomic vacancies and the increasing and relaxing of surface strain submicron defects via, respectively, quenching and annealing. The formation of these vacancies and defects can only be speculated due to the limitation of equipment for their experimental detection was not obtainable. In future works, the effect of defect could be potentially used and expanded the magnetic or dielectric properties to other ferrite materials since the effect has been proven to control the desired properties value. The empirical recovery pattern is clearly revealed for the industrial and technological usefulness even though the fundamental scientific explanation might not be completely precise.
